# Platelets’ Nanomechanics and Morphology in Neurodegenerative Pathologies

**DOI:** 10.3390/biomedicines10092239

**Published:** 2022-09-09

**Authors:** Velichka Strijkova, Svetla Todinova, Tonya Andreeva, Ariana Langari, Desislava Bogdanova, Elena Zlatareva, Nikolay Kalaydzhiev, Ivan Milanov, Stefka G. Taneva

**Affiliations:** 1 Institute of Biophysics and Biomedical Engineering, Bulgarian Academy of Sciences, “Acad. G. Bontchev” Str. 21, 1113 Sofia, Bulgaria; 2 Institute of Optical Materials and Technologies “Acad. Yordan Malinovski”, Bulgarian Academy of Sciences, “Acad. G. Bontchev” Str. 109, 1113 Sofia, Bulgaria; 3 Faculty of Applied Chemistry, Reutlingen University, Alteburgstraße 150, D-72762 Reutlingen, Germany; 4 Department of Neurology, University Multiprofile Hospital for Active Treatment in Neurology and Psychiatry Sv. Naum, 1113 Sofia, Bulgaria

**Keywords:** neurodegenerative disorders, platelets, atomic force microscopy, surface roughness, stiffness, area, height

## Abstract

The imaging and force–distance curve modes of atomic force microscopy (AFM) are explored to compare the morphological and mechanical signatures of platelets from patients diagnosed with classical neurodegenerative diseases (NDDs) and healthy individuals. Our data demonstrate the potential of AFM to distinguish between the three NDDs—Parkinson’s disease (PD), amyotrophic lateral sclerosis (ALS) and Alzheimer’s disease (AD), and normal healthy platelets. The common features of platelets in the three pathologies are reduced membrane surface roughness, area and height, and enhanced nanomechanics in comparison with healthy cells. These changes might be related to general phenomena associated with reorganization in the platelet membrane morphology and cytoskeleton, a key factor for all platelets’ functions. Importantly, the platelets’ signatures are modified to a different extent in the three pathologies, most significant in ALS, less pronounced in PD and the least in AD platelets, which shows the specificity associated with each pathology. Moreover, different degree of activation, distinct pseudopodia and nanocluster formation characterize ALS, PD and AD platelets. The strongest alterations in the biophysical properties correlate with the highest activation of ALS platelets, which reflect the most significant changes in their nanoarchitecture. The specific platelet signatures that mark each of the studied pathologies can be added as novel biomarkers to the currently used diagnostic tools.

## 1. Introduction

Platelets, anucleated circulating blood cells, function as regulators of hemostasis and thrombosis, and are involved in inflammatory processes and pathological conditions, which are risk factors for neurodegenerative diseases (NDDs) [1,2,3]. They also play a role in immunity and communication with other cells and tissues [3]. 

Platelets contain a large number of bioactive molecules, stored in dense and alpha granules that are secreted upon platelet activation and mediate their function [4,5], and glycogen granules that are the source of energy for platelet interactions [2]. The dense granules contain small molecules, such as ADP, ATP, polyphosphate, serotonin and calcium [6]. The alpha granules are a storage pool for proteins such as fibrinogen, coagulation and growth factors, adhesive molecules, cytokines and chemokines [7]. 

Importantly, platelets exhibit structural and biochemical characteristics similar to neurons [8]; the platelet granules resemble the neuron vesicles [9,10], and hence they have been considered as a good peripheral model to study neurodegenerative pathologies [1,11,12]. In various NDDs, including Alzheimer’s disease (AD), amyotrophic lateral sclerosis (ALS) and Parkinson’s disease (PD), platelets experience activation and aggregation [13]. Furthermore, oxidative and physiological stress induce structural and functional alterations and activation of platelets in a variety of NDDs, including AD, ALS and PD [13,14,15,16,17], in the manner of those observed with aging [14]. 

The contribution of platelets in NDDs has been most extensively studied for AD; however, their exact role is still debated (see the review of Ferrer-Raventós et al. [18]). They are the major source of amyloid transmembrane protein precursor (APP) [19,20,21,22,23] and provide a large percentage of amyloid β peptide (Aβ) in blood plasma. The concentration of APP isoforms in platelets is comparable to those in the brain but with a different expression pattern [20]. APP is thought to act as a receptor on the platelet surface and is also crucial for the regulation of intracellular Ca^2+^ concentration [24]. Aβ was found in dense platelet microvesicle fractions and can be secreted upon platelet activation [11]. The secreted Aβ peptide variants are as those found in the senile plaques of patients with AD [25]; however, platelets mainly release the Aβ(1-40) peptide, while neurons produce high amounts of Aβ(1-42) peptides [20].

Some authors, however, suggest the opposite, namely, that circulating Aβ originates in the central nervous system, crosses the blood–brain barrier and can be absorbed by platelets and other blood cells [20,26]. Regardless of its origin, produced either by platelets or by brain, Aβ affects their functions. Studies of AD platelets show changes in the operation of APP, in membrane fluidity, cholesterol, serotonin uptake and intracellular levels of Ca^2+^. A correlation between changes in the ratio of different APP isoforms in platelets and a decline in the cognitive skills of patients in the early stages of AD was found, suggesting that the ratio of APP isoforms in platelets is a biomarker for the early AD stage [27]. Family mutations of AD lead to the hyperactivation of circulating platelets, which is evident with the progression of the disease. The vascular damage observed in AD is a natural cause of platelet activation and degranulation [28].

Platelets from patients with PD and ALS are also characterized by abnormal features. In PD, the disordered protein α-synuclein is associated with changes in platelet morphology and is overexpressed, and found in microvesicles [29,30]. The pathology is accompanied by the hyperactivation and granulation of platelets and the significant production of reactive oxygen species [31]. There is also evidence for changes in the ultrastructure of these cells, mitochondrial dysfunction and elevated glutamate levels in PD [32].

It has been shown that not only motor neurons, but platelets are also affected in ALS [33]. The distinct characteristics of ALS platelets, such as the heterogeneous distribution of platelet granules, formation of vacuoles, vesicles, pseudopods formation, weak differentiation of the cell membrane and substantially increased surface area, were reported [34,35]. The DNA-binding protein TDP-43, a pathological protein in motor neurons of sporadic ALS, has a significantly higher concentration in ALS platelets than in healthy ones, and its location in the nucleus is modified and it is relocated from the nucleus to cytoplasmic inclusions in ALS patients [33]. Importantly, Shrivastava et al. [34,35] identified ultrastructural platelet modifications in ALS cases, and mitochondrial abnormalities (changes in the permeability and potential of the mitochondrial membrane) and dysfunction [36,37]. The etiological factors responsible for ALS are also related to platelets [34]. 

Furthermore, the reduced serotonin level, found in ALS and in AD platelets, reflects changes in the neurons of the two pathologies [38,39,40]; the lower serotonin level was shown to correlate with ALS patient survival [38].

In this work, we employ atomic force microscopy (AFM) to characterize the platelet topography, morphology, nanomechanics and activation state in the neurodegenerative disorders PD, ALS and AD, searching for new diagnostic biomarkers. Our findings provide evidence for strongly altered roughness, stiffness and the activation state of platelets that can differentiate between the studied pathologies.

## 2. Materials and Methods

### 2.1. Studied Individuals

The study included 25 patients diagnosed with NDDs. A total of 11 idiopathic patients with PD (4 males and 7 females, mean age 64.6 ± 3.1 (47 to 79 years)) were diagnosed according to the 2015 MDS-PD clinical criteria [41]. All were with bilateral motor symptoms, and the median Hoehn and Yahr stage was 3 (HY range II–IV) [42]. Patients with comorbid dementia were excluded. A total of 9 patients with ALS (5 males and 4 females, 59.0 ± 4.2 (42 to 78 years)) were selected based on the El Escorial criteria [43], 6 were with clinically definite and 3 with clinically probable and laboratory supported forms of ALS. The mean severity score was 34.5 (mild to moderate) according to the Revised ALS Functional Rating Scale (ALSFRS-R) [44]. A total of 5 patients with probable AD (5 females, mean age 76.0 ± 3.0 (70 to 83 years)) who fulfilled DSM-IV criteria for mild to moderate AD were enrolled [45]. Mini-Mental State Examination score was 17 and 21 [46]. Patients with depression were not included. 

In the control group, 9 healthy individuals (6 females and 3 males, mean age 64.0 ± 3.1 years (42 to 71 years)) were included; none of them had a history of any neurodegenerative, hereditary burden or another disease, and any treatment. 

This study was approved by the ethics committee of the University multiprofile hospital for active treatment in neurology and psychiatry “St. Naum” (UMHATNP), Sofia, (Consent number 05/15.03.2018) and was conducted in agreement with the principles of the Declaration of Helsinki of 1975, revised in 2013 for research involving human subjects. All patients signed an informed written consent prior to blood collection.

### 2.2. Isolation and Immobilization of Platelets

Platelets were isolated according to Protocol 9 in [47] from fresh venous blood, 5 mL, drawn from patients with NDDs and healthy volunteers using a 19-gauge needle, and transferred in ethylene diamine-tetra acetic acid (EDTA) vacutainers (0.084 mL 15% EDTA Becton, Dickinson and Company, NJ, USA). The blood was centrifuged at 150× *g* for 15 min at room temperature, and the platelet-rich supernatant was centrifuged at 390× *g* for 5 min. The platelet-poor supernatant was carefully removed and the pellet containing platelets was gently resuspended in PBS buffer, pH 7.2, and centrifuged at 100× *g* for 5 min. 

For AFM imaging, the platelet suspension was deposited onto a sterilized glass coverslip and, after 30 min of incubation, the loosely adhered platelets were removed by rinsing with PBS. The adhered platelets were then fixed with 1% glutaraldehyde (pH 7.4) and rinsed (three times) with PBS.

### 2.3. AFM Imaging and Force Mapping

Platelet imaging and force mapping were performed by means of a commercial atomic force microscope (MFP-3D, Asylum Research, Oxford Instruments, Santa Barbara, CA 93117, USA) at room temperature in contact mode using silicon nitride probes (type qp-Bio, Nanosensors) with a spring constant of 0.06 N/m, resonant frequency 16 kHz, conical shape and nominal tip radius of 8 nm. The AFM images were analyzed using Gwyddion-2.57 and IgorPro 6.37 software to determine the area (A), height (H) and surface roughness (root-mean-square surface roughness, R_rms_) of the platelets. Images of 98 cells from healthy individuals, 134 cells from PD, 106 from ALS and 80 from AD were analyzed.

Platelet roughness was determined over an area of 0.5 × 0.5 µm, localized in the center of the cells after the preliminary leveling of the surface to avoid the influence of the spherical profile. R_rms_ was calculated according to the equation [48,49]:

(1) $$R_{\mathrm{rms}}=\sqrt{\sum_{i=1}^{N}\frac{{\left(Zi-Zn\right)}^{2}}{\left(N-1\right)}}$$

where *N* is the total number of points, *Z_i_* is the height of the *i*-th point and *Z_n_* is the average height.

The force mapping was performed on a grid of 32 × 32 points. The images were collected at a scanning speed of ca. 2 s/row. Before the measurements, the tip was calibrated on a clean glass substrate using special software Igor Pro.

The Young’s modulus (*E_a_*) was determined by analyzing the force–distance curves applying the Hertz model [50,51]:

(2) $$F(\delta )=\frac{2E_{a}tan(\alpha )}{\pi \left(1-v^{2}\right)}$$

where ***E_a_*** corresponds to the apparent Young’s modulus, *υ* is the Poisson ratio and *δ* is the indentation depth.

A non-parametric statistical test was performed using OriginPro 2018 software. The difference in the R_rms_, A, H and ***E_a_*** values of platelets from the NDD groups vs. the healthy group was considered statistically significant for *p* < 0.05.

### 2.4. Correlation Analysis

Pearson’s correlation analysis was performed to the dataset of AFM parameters derived for platelets from patients diagnosed with PD, ALS and AD, and healthy individuals. The correlation coefficient, corr(*X*, *Y*), where *X* and *Y* are two parameters that characterize the studied platelets, was determined according to the equation:

(3) $$r=corr\left(X,Y\right)=\frac{co\nu \left(X,Y\right)}{\sigma \left(X\right)\cdot \sigma \left(Y\right)}$$

where *coν*(*X*, *Y*) denotes the covariance of *X* and *Y*, while *σ(X)* and *σ(Y)* are the corresponding standard deviations.

If n observations were made (*x*(1), *x*(2),…, *x*(*n*) of *X* and *y*(1), *y*(2),…, *y*(*n*) of *Y*), the covariance and standard deviations for Pearson’s correlation were evaluated according to the following equations:

(4) $$co\nu \left(X,Y\right)=\frac{1}{n-1}\overset{n}{\underset{i=1}{\sum }}\left(x\left(i\right)-\tilde{x})\;y(\left(i\right)-\tilde{y}\right)$$

(5) $$\sigma \left(Z\right)=\frac{1}{n-1}\overset{n}{\underset{i-1}{\sum }}{\left(z\left(i\right)-\tilde{z}\right)}^{2}$$

The *corr(X, Y)* values are in the interval [−1, 1], where the boundary values (±1) are obtained in the case of normal distributions and a linear dependence between the quantities. 

## 3. Results

### 3.1. Topography and Morphology of Human Platelets from PD, AD and ALS Patients and Healthy Individuals

**Figure 1:**
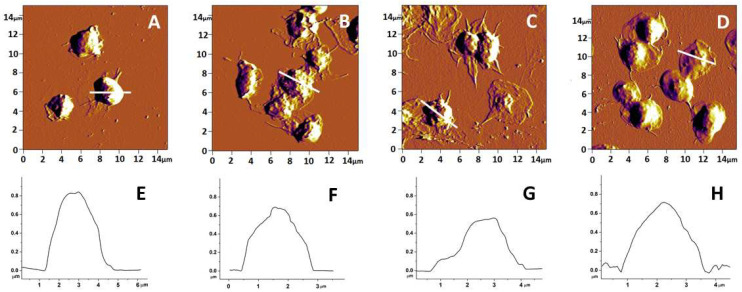
AFM images of platelets from healthy individuals (**A**) and patients with PD (**B**), ALS (**C**) and AD (**D**), and cross-sectional profiles (**E**–**H**) corresponding to the transversal white lines in panels (**A**–**D**).

Representative AFM-images of platelets isolated from patients with the three studied pathologies (PD, ALS and AD) and from healthy controls are shown in Figure 1. It is clearly seen that the adhered platelets from healthy individuals have a typical for the resting state discoid shape with a hemisphere profile, and are poorly activated (Figure 1A,E). In healthy platelets, pseudopods are rarely seen, and if seen, they are short and poorly formed. Neither platelet clustering nor formation of lamellipodia were observed (Figure 1A). 

Four stages of platelet activation were observed during the process of in vitro adhesion. These are: (I) adhesion of resting platelets; (II) development of pseudopods; (III) formation of both pseudopods and lamellipodia; and (IV) presence of widespread lamellipodia without pseudopods [52,53]. In the three NDD groups of patients, the platelet morphology was significantly changed. In PD, platelets had an altered, asymmetric profile (Figure 1B,F) and clearly developed pseudopods, which is typical of an advanced stage of activation, generally stage II; only a few platelets were in stage III. Clusters of cells were also distinguished (Figure 1B). An even more advanced activation was found for platelets from the ALS group of patients, which were in activation stage III and IV. The platelet shape was strongly altered, asymmetrical and in most cases of “fried egg” type (Figure 1C,G). The actin cytoskeleton of ALS platelets underwent strong rearrangement with the advanced formation of pseudopods, as well as broad lamellipodia. Pseudopods were strongly intertwined between cells, together with clearly delineated outflow of hyaloplasm widely around the platelets (Figure 1C). In contrast to the PD and ALS groups, platelets isolated from patients with AD showed a relatively preserved spherical shape and symmetrical profile similar to that of control platelets (Figure 1D,H). It appears that AD platelets own a specific activation state resembling state II but with a weak outflow of hyaloplasm around the cells, in most cases with a lack of pseudopods (Figure 1D). 

Platelet height and area in patients with NDDs differed significantly from those of healthy individuals, with values of both parameters being lower for NDD than for healthy platelets and decreasing in the following order: healthy > PD ≥ AD > ALS (Table 1). The most drastic effect on the platelet morphology was observed in the ALS pathology—the height was twice lower and the area 1.4 times lower than in normal healthy state. For PD and AD, the effect was weaker—the reduction in height was 1.87 times for PD and only 1.2 for AD platelets, and about 1.1–1.2 for the area of PD and AD platelets, respectively (Table 1).

The morphometric parameters, height (H), area (A), and roughness (R_rms_), as well as the stiffness (E_a_) of the platelet membrane (discussed in the next section), are summarized in Table 1.

**Figure 2:**
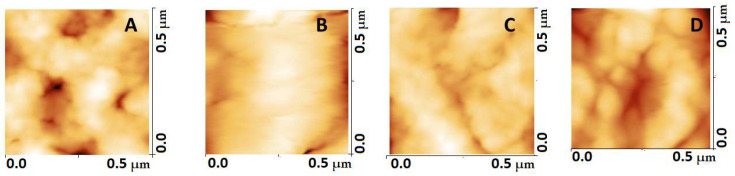
Comparison of the ultrastructure of the platelet membrane in healthy individuals (**A**), patients with PD (**B**), ALS (**C**) and AD (**D**). The images were taken in the middle part (0.5 × 0.5 µm) of the platelets.

The membrane of pathological platelets underwent significant alterations compared to the control ones. The membrane roughness was drastically affected, demonstrating lower values in the studied NDDs than in healthy platelets (Table 1). The decrease in R_rms_, accompanying the membrane smoothening, was much more pronounced for PD than for ALS and AD platelets (Table 1). Figure 2 offers some ultrastructural insights into the internal morphology of control and NDD platelet membranes. The membrane of control platelets was evenly folded (Figure 2A) over the entire surface. Platelets from PD and ALS patients showed a highly smoothed structure (Figure 2B,C), while those of AD patients were characterized by a more folded membrane compared to PD and ALS, and smoother structural formations were seen in some areas (Figure 2D). It should be noted, however, that the difference between the heights of the highest and lowest points of the NDD platelet membrane is smaller compared to the controls (healthy > AD > ALS > PD), which determines their lower R_rms_ value (Table 1). 

### 3.2. Nanomechanical Characteristics of Platelets from NDD Patients

**Figure 3:**
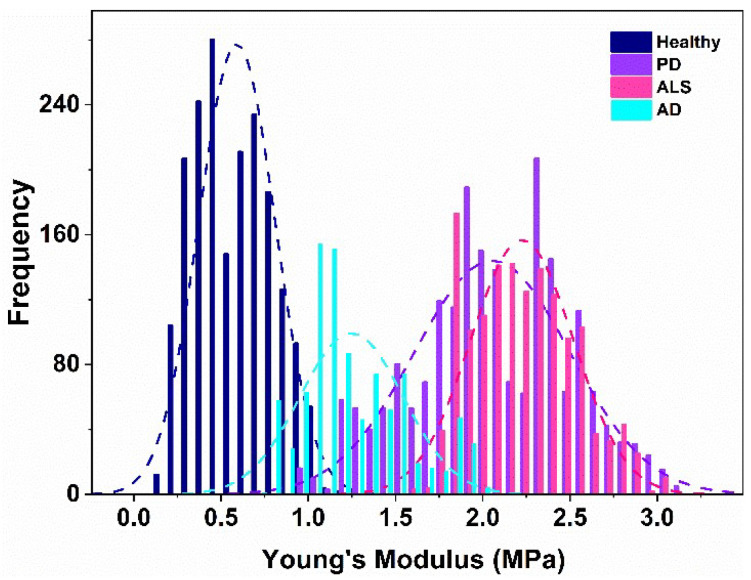
Histograms of the distribution of the Young’s modulus and the corresponding Gaussian distributions of the stiffness of platelets from healthy individuals (blue) and patients with AD (cyan), PD (violet) and ALS (pink).

The platelet stiffness (*E_a_*) was calculated from the force–distance curves using the Hertz model. The histograms of the *E_a_* distribution for the studied groups of patients and the healthy individuals are presented in Figure 3.

The values of the Young’s moduli *E_a_* in this work are in the range of 0.60–2.18 MPa that correspond well to those obtained by Li et al. [54]. Previous AFM experiments demonstrated a strong difference in Young’s modulus of fixed/dehydrated (as in this work) and unfixed/hydrated platelets, which are 30–130 MPa and 1–50 kPa, respectively [54,55,56,57].

**Figure 4:**
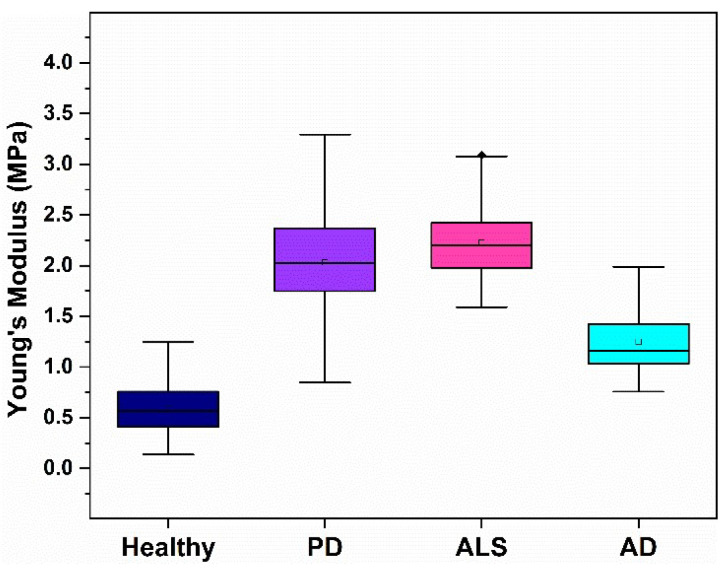
Young’s moduli of platelets from the healthy individuals and patients with PD, ALS and AD pathologies.

The membrane stiffness of NDD platelets drastically differed from that of the healthy ones, especially for the patients with PD and ALS (Table 1). The stiffening effect caused by AD was less significant compared to that induced by both PD and ALS (healthy < AD < PD < ALS), the mean *E_a_* value was 2 times higher for AD, and 3.4–3.6 times for PD and ALS, respectively, relative to healthy platelets (Figure 4, Table 1).

### 3.3. Correlation Analysis of Platelets’ Nanomechanical and Morphological Parameters

**Figure 5:**
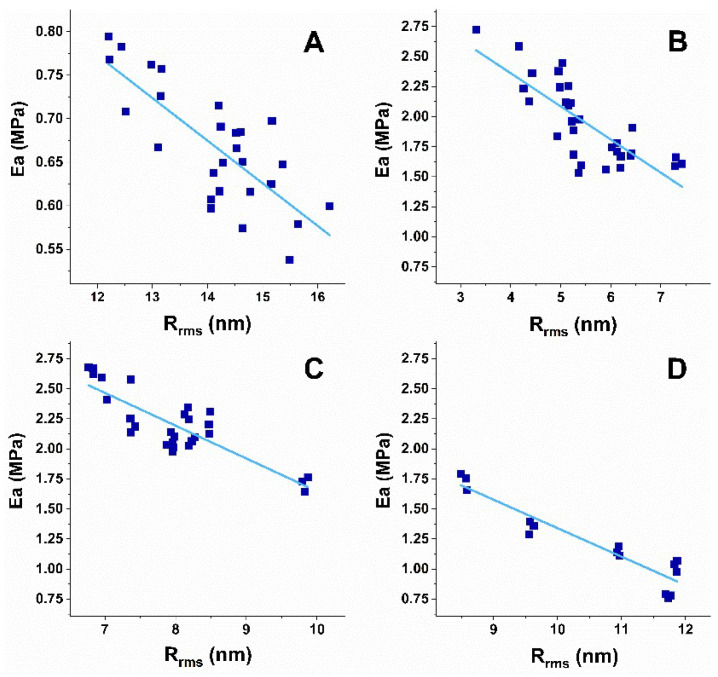
Scatter plots of *E_a_* against R_rms_ for platelets derived from the healthy individuals (**A**) and the PD (**B**), ALS (**C**) and AD (**D**) patients.

**Figure 6:**
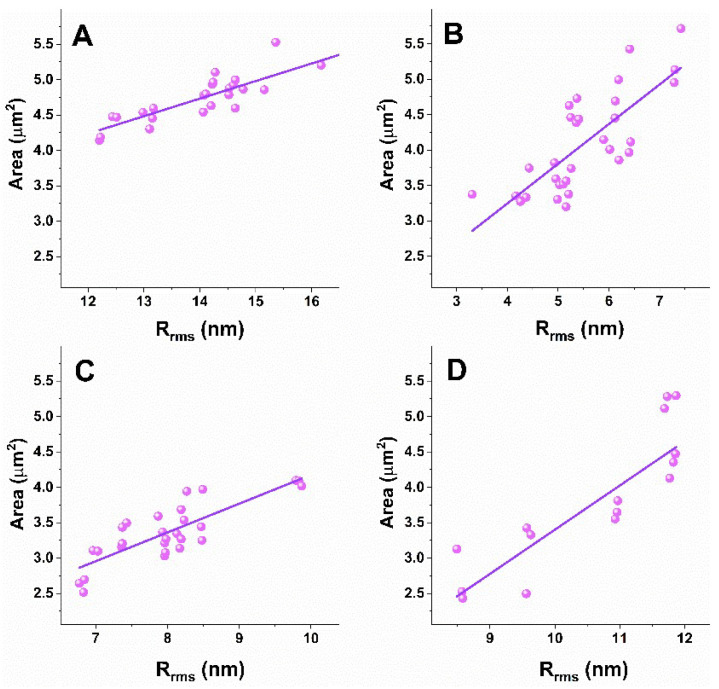
Scatter plots of Area against R_rms_ for the platelets derived from the healthy individuals (**A**) and the PD (**B**), ALS (**C**) and AD (**D**) patients.

A correlation approach was applied to analyze the nanomechanical and morphological parameters of platelets derived from patients with the studied NDDs and healthy individuals, and to determine correlations between the different parameters. The evaluated Pearson’s correlation coefficients (r) are presented in Table 2 and graphically in scatter plots for two platelets parameters pairs, *E_a_*/R_rms_ in Figure 5 and Area/R_rms_ in Figure 6. No correlation was found for the other pairs of parameters.

The analysis shows a strong negative correlation for the *E_a_*/R_rms_ (Figure 5) and a strong positive correlation for the Area/R_rms_ (Figure 6) pairs for NDD and healthy platelets. The different degree of the relation between *E_a_* and R_rms_ was proven for healthy and pathological platelets, the strongest one for AD ones (Table 2). The degree of the Area and R_rms_ relation is similar for the healthy, ALS and AD platelets, and lower for PD ones (Table 2). The narrow 95% confidence interval proves a strong relation between both pairs of parameters and accuracy of the AFM data. Accordingly, the coefficient of determination (r^2^), a measure of the strength of correlation, is >0.5 for the healthy and NDD platelets for both *E_a_*/R_rms_ and Area/R_rms_, indicating that the included data represent strong prediction sets. For the *E_a_*/R_rms_ parameters, r^2^ indicates a very good result for the ALS and AD groups (r^2^ = 0.7 and r^2^ = 0.86, respectively), and good results for healthy and PD (r^2^ = 0.56 and r^2^ = 0.6, respectively), while for the Area/R_rms_ pair, r^2^ shows very good results for the healthy and AD (r^2^ = 0.77 and r^2^ = 0.72, respectively) sets and a good result for PD and ALS (r^2^ = 0.56 and r^2^ = 0.66, respectively) (Table 2).

In summary, the correlation coefficients and the coefficient of determination prove that the strength of relation between the *E_a_*/R_rms_ pair changes in the order AD > ALS > PD > healthy, while the strength of relation for the Area/R_rms_ pair in the order healthy ≅ ALS ≅ AD > PD platelets.

## 4. Discussion

Anucleate human blood platelets are a crucial component in maintaining hemostasis to prevent excessive blood loss from a vascular damage and play a key role in inflammation and immunity. In this work, AFM was explored to determine the morphometric and mechanical signatures of platelets derived from patients with the classical neurodegenerative pathologies PD, ALS and AD. The biomechanical properties of platelets are highly relevant for their function. These properties are provided mainly by the cytoskeleton, which governs morphological and biochemical changes and control the shape, secretion of granular contents, degree of activation, deformation, aggregation and spreading (during adhesion) of platelets and, therefore, can potentially serve as label-free diagnostic markers of platelet-related pathologies. We found significant differences between the NDD and healthy platelet signatures. The three pathologies were associated with activation-related shape changes expressed in a decrease in the platelets’ lateral dimensions and height and in the membrane smoothing reflected in the reduction in the membrane surface roughness. Furthermore, platelets isolated from patients with NDDs contrasted with those of the healthy ones in enhanced membrane stiffness. These differences were more apparent in ALS and PD than in AD platelets. Importantly, the platelet parameters were affected to a different extent in the three pathologies, thus allowing parameter-specific differentiation between ALS, PD and AD. Area, height and stiffness were the most affected in ALS, while the membrane surface roughness in PD patients and all parameters were less influenced in AD patients. It is to be noted that, in contrast to a reduction in the size and membrane roughness of the NDD platelets, platelets from patients with acute myocardial infarction were found to have a larger diameter and height, as well as increased roughness compared to normal platelets [54]. It is also intriguing that, unlike the NDDs studied in this paper, a familial case study reported the presence of giant platelets in the peripheral blood of a family with May–Hegglin anomaly associated with familial spastic paraplegia [58]. Hereditary spastic paraplegia (HSP) is another group of genetic neurodegenerative diseases characterized by the presence of progressive lower limb spasticity and weakness [59]. The common pathological feature is the retrograde degeneration of the longest nerve fibers in the corticospinal tracts and posterior columns [60]. More than 90 different genetic types were determined [61] and the genetic map, “HSPome”, showing the interaction between the HSP-associated genes, was created [62]. Importantly, the similarities and overlapping of some genes implicated in the common NDDs—ALS, AD and PD—which were studied in this paper, and HSP have been reported [62,63]; especially type 11 HSP (with mutation in SPG11 gene) showed pathological similarities with ALS [64], while a small percentage of HSP patients displayed PD-specific symptoms in the dopamine transmission impairment [65]. In this context, further studies focused on HSP patients exploring AFM should provide additional information and might be helpful for differentiating HSP from ALS and other disorders.

Both the shape and the activation state were most strongly modified in ALS and least in AD platelets, in line with previous reports on the relation between the platelets’ shape and their activation state [3]. Moreover, ALS platelets appeared “fried egg”-shaped and asymmetrical in contrast to the typical flat discoid shape characteristic of resting healthy platelets. Additionally, an advanced stage of pseudopod formation was observed in ALS, while almost no pseudopods were formed in healthy cells. Furthermore, the changes in PD platelets, although less pronounced, followed the same trend as those in ALS cells. More pronounced cell activation and clustering was found in both ALS and PD over AD platelets. ALS platelets were distinguished from the others by having the smallest area and height, while the PD ones had the smoothest membrane. Shrivastava et al. [34] have also revealed pseudopodia formation, platelet activation and morphological changes in ALS platelets by means of transmission electron microscopy. The different extent of morphological modifications in platelets from the three studied pathologies might be attributed to the expression of different pathological proteins and different proteins exposed on the platelet surface. In particular, α-synuclein is overexpressed in PD platelets [30,66] and detected in less dense microvesicles [30]; amyloid β peptide fibrils are stored in dense platelet microvesicles in AD [29]; higher levels of TDP-43 protein [33] and lower serotonin level [34,38] were detected in ALS platelets. 

Another common feature of the NDD platelets revealed in this paper, which can be used as a quantitative indicator for NDDs, was that they were mechanically enhanced compared to the healthy cells. The increase in the elastic modulus was most significant for ALS and PD (3.6 and 3.4 times compared to the healthy platelets) and less for AD platelets (2 times compared to the healthy platelets). This suggests more significantly modified platelet cytoskeleton in ALS and PD than in AD, considering previous evidence that both the elastic properties and the ultrastructure of the platelet membrane are strongly related to the platelet cytoskeleton, activation and aggregation state, accompanied by the presence of different misfolded proteins/peptides [53,67,68,69,70]. The highest stiffness corresponds to the highest activation of the ALS platelets in agreement with the previous data on collagen-stimulated platelet activation related to increased stiffness [54]. On the contrary, softer platelets are characteristic of other pathologies, such as acute myocardial infarction patients [54,71], cancer and metastatic cells in comparison with normal healthy cells [72,73,74,75,76,77]. 

Furthermore, since PD, ALS and AD are age-related disorders, it is worth proving that the observed alterations in the platelets’ biophysical features are disease- and not age-induced. It has been found that aging accompanies many processes at the cellular and molecular levels [78], including the activity, aggregation capacity and cytoskeletal organization of platelets [14,79]. Several studies reported a drop in platelet counts in individuals older than 70 years, and stable counts in young–middle aged individuals [78,80,81,82]. In contrast, no age-related decline was found in platelet count either for males or females in another investigation [83]. A similar platelet count was found between PD and AD groups of patients [84], as well as between AD patients and age-matched controls, although AD platelets were in a more activated state [85]. It was also reported that platelets from middle-aged (41–72 years old) individuals aggregate more significantly compared to young individuals (21–30 years old) [79]. An age-related decrease in platelet membrane regularity and smoothness, accompanied with multiple platelet membrane ruptures (at age over 65 years), is evidenced by electron tomography in a very recent study as opposed to the increase in smoothness reported in this paper [86]. Platelet size was found to increase during the course of life [87], contrary to the pathology-related size decrease observed in this study. In this context, we did not notice strict dependences of the studied parameters on the age of patients in the age range of the NDD groups as well as in the group of healthy individuals, most probably because they belong to the same middle–old-aged individuals. Therefore, we can safely attribute the observed differences in platelets’ biophysical parameters to the particular disorder and not to the age of the patients.

Similar to platelets, our previous investigation has shown strong modifications of the morphological and nanomechanical features of red blood cells from patients with the three neurodegenerative pathologies, namely, lower surface roughness and higher stiffness as well as a reduced abundance in the typical biconcave discoid shape compared to healthy red blood cells [88]. Hence, the biophysical parameters of red blood cells are modified in a similar manner as platelets that might reflect cytoskeleton alterations in both peripheral blood cells presumably caused by the same pathological proteins/peptide for each pathology.

Therefore, our results on platelets biophysical characteristics of NDDs and on other diseases, mentioned above, point to the strong disease specificity of platelet stiffness and morphological signatures. 

## 5. Conclusions

It is becoming increasingly clear that severe pathologies are associated with changes in the shape, size, membrane structure and elasticity of platelets, which, in turn, affect the ontogenesis/biogenesis and function of these cells. Here, we demonstrated that NDDs exert a strong and specific impact on the morphometric and the mechanical parameters of human platelets. Common features of the three studied neurodegenerative pathologies are the lower membrane surface roughness, area and height, and the significantly higher stiffness, in addition to the different degree of activation, distinct pseudopodia and nanoclusters formation. ALS has the strongest impact on platelet signatures; the largest stiffness correlates with the highest activation, which results from most significant changes in the platelets’ nanoarchitecture and, most probably, reflects the higher aggressiveness of this pathology than that of PD and AD.

Overall, our results imply that the mechanical and morphological properties of platelets distinguish the three NDDs from the healthy normal state and, moreover, differentiate ALS, PD and AD among each other. The modulated nanomechanics are strongly related to NDDs-induced alteration in the cytoskeleton of platelets that plays a key role in all their functions.

## Figures and Tables

**Table 1:** Morphometric (H, A and R_rms_) and nanomechanical (*E_a_*) parameters of platelets from patients with NDD and healthy individuals determined from the AFM images and force–indentation curves, mean values and SD.

Subject of Investigation	Area (μm^2^)	Height (μm)	Roughness (nm)	*E_a_* (MPa)
Healthy	4.8 ± 0.5	1.00 ± 0.18	14.3 ± 2.2	0.60 ± 0.21
PD	4.2 ± 0.6	0.53 ± 0.12 **	5.4 ± 1.2 **	2.04 ± 0.36 *
ALS	3.4 ± 0.9 *	0.50 ± 0.17 **	8.0 ± 0.9 **	2.22 ± 0.33 *
AD	4.0 ± 1.1	0.82 ± 0.10	10.5 ± 1.4	1.25 ± 0.29 *

*p*-values < 0.05 are denoted by *; *p*-values < 0.01 are denoted by **.

**Table 2:** Pearson’s correlation coefficient, r, calculated for the parameter pairs *E_a_*/R_rms_ (Young’s modulus and membrane roughness) and Area/R_rms_ (platelet’s area and membrane roughness), for healthy and NDD platelets. The 95% confidence intervals (CI) for Pearson’s correlation are provided.

Subject of Investigation	*E_a_*/R_rms_	CI		Area/R_rms_	CI	
	r	Lower Limit	Upper Limit	r	Lower Limit	Upper Limit
Healthy	−0.75	−0.8794	−0.5175	0.88	0.7512	0.9442
PD	−0.78	−0.8887	−0.5882	0.76	0.5552	0.8779
ALS	−0.84	−0.9248	−0.6757	0.81	0.6212	0.9099
AD	−0.93	−0.9769	−0.7978	0.85	0.5982	0.949

## Data Availability

The data that support the findings of this study are available from the corresponding author upon reasonable request.
